# Differential Epigenetic Status and Responses to Stressors between Retinal Cybrids Cells with African versus European Mitochondrial DNA: Insights into Disease Susceptibilities

**DOI:** 10.3390/cells11172655

**Published:** 2022-08-26

**Authors:** Shari R. Atilano, Sina Abedi, Narcisa V. Ianopol, Mithalesh K. Singh, J Lucas Norman, Deepika Malik, Payam Falatoonzadeh, Marilyn Chwa, Anthony B. Nesburn, Baruch D. Kuppermann, M. Cristina Kenney

**Affiliations:** 1 Gavin Herbert Eye Institute, Ophthalmology Research Laboratory, University of California Irvine, Hewitt Hall, Room 2028, 843 Health Science Rd., Irvine, CA 92697, USA; 2 Cedars-Sinai Medical Center, Los Angeles, CA 90048, USA; 3 Department of Pathology and Laboratory Medicine, University of California Irvine, Irvine, CA 92697, USA

**Keywords:** mitochondrial DNA, amyloid β, UV radiation, cybrids, epigenetics, maternal African-origin mtDNA, European-origin mtDNA, haplogroup, disease susceptibility

## Abstract

Mitochondrial (mt) DNA can be classified into haplogroups, which represent populations with different geographic origins. Individuals of maternal African backgrounds (L haplogroup) are more prone to develop specific diseases compared those with maternal European-H haplogroups. Using a cybrid model, effects of amyloid-β (Amyβ), sub-lethal ultraviolet (UV) radiation, and 5-Aza-2′-deoxycytidine (5-aza-dC), a methylation inhibitor, were investigated. Amyβ treatment decreased cell metabolism and increased levels of reactive oxygen species in European-H and African-L cybrids, but lower mitochondrial membrane potential (ΔΨM) was found only in African-L cybrids. Sub-lethal UV radiation induced higher expression levels of *CFH*, *EFEMP1*, *BBC3*, and *BCL2L13* in European-H cybrids compared to African-L cybrids. With respect to epigenetic status, the African-L cybrids had (**a**) 4.7-fold higher total global methylation levels (*p* = 0.005); (**b**) lower expression patterns for *DNMT3B*; and (**c**) elevated levels for *HIST1H3F*. The European-H and African-L cybrids showed different transcription levels for *CFH*, *EFEMP1*, *CXCL1*, *CXCL8*, *USP25*, and *VEGF* after treatment with 5-aza-dC. In conclusion, compared to European-H haplogroup cybrids, the African-L cybrids have different (**i**) responses to exogenous stressors (Amyβ and UV radiation), (**ii**) epigenetic status, and (**iii**) modulation profiles of methylation-mediated downstream complement, inflammation, and angiogenesis genes, commonly associated with various human diseases.

## 1. Introduction

Mitochondria (mt) are unique organelles with their own circular DNA. The mtDNA-coding region encodes for 37 genes including 13 protein subunits critical for oxidative phosphorylation (OXPHOS), 22 tRNAs, and 2 rRNAs. The non-coding control region (MT-Dloop), contains 1121 nucleotides and is important for replication and transcription [1,2,3]. mtDNA can be categorized into haplogroups defined by single-nucleotide polymorphism (SNP) variants, which represent the different geographic origins of populations. Mitochondria are known to play a critical role in OXPHOS, but recent studies have provided evidence that mtDNA haplogroups can also influence the expression of genes related to oxidative stress [4]. Further investigations report that mtDNA haplogroups/variants can either be protective or high risk for a number of diseases, including age-related macular degeneration (AMD), glaucoma, cancers, diabetes, Alzheimer’s disease, Parkinson’s disease, and cardiomyopathies [4,5,6,7,8,9,10,11,12,13,14,15,16]. Greater clarification on the retrograde signaling (mitochondria to nucleus) has become evident through the use of cybrids, which are cell lines with identical nuclei but mtDNA from different individuals. These findings support the concept that the mtDNA haplogroups play a role in responses to oxidative stressors.

Oxidative stress plays a significant role in the development and progress of diseases. For example, the accumulation of amyloid-β (Amyβ) is associated with Alzheimer’s disease (extracellular Aβ plaques), age-related macular degeneration (drusen), cardiomyopathy, and diabetes (islet amyloid aggregation) [17,18,19,20,21,22,23,24]. In all of these diseases, the accumulation of Amyβ is cytotoxic and contributes to the pathology. The risk of developing Alzheimer’s disease and related dementias (ADRDs) is higher in diabetic subjects compared to non-diabetics [25,26]. Another source of oxidative stress that contributes to disease is ultraviolet (UV) radiation, which has been associated with retinopathies, skin cancers, and decreased longevity [27]. The roles that mitochondria play in the responses to Amyβ and UV radiation are not clear. Using the transmitochondrial cybrid model, studies have shown that cybrids containing the H versus the J mtDNA haplogroup profiles show different responses to UV radiation [28] and cisplatin [29], while H cybrids versus K cybrids are different in their response to Amyβ peptides [30]. To date, the differential responses to these stressors have not been investigated in cybrids possessing maternal European mtDNA haplogroups versus maternal African haplogroups.

Transmitochondrial cybrids have also been used to investigate the mitochondrial–nuclear interactions for various diseases. In a previous study, it was demonstrated that cybrids with maternal African-origin mtDNA (L haplogroup) had significantly different expression levels for complement, inflammation, and apoptosis pathway genes compared to cells with common European-origin mtDNA (H haplogroup) [31]. Additionally, Dolinko et al. suggest that mitochondria from African and Asian diabetic subjects possess a “metabolic memory” that confers resistance against hyperglycemia, hypoxia, and demethylation, and that this “metabolic memory” can be transferred into the RPE (retinal pigment epithelium) cybrid cell lines in vitro [32]. However, the signal transduction mechanism(s) involved with mitochondria-to-nuclear signaling are not fully understood. Recent studies have shown an association between mitochondria and the methylation status of cells. When cells were depleted of their mitochondria, the degree of DNA methylation was affected [33]. S-adenosylmethionine formation and mitochondrial functions are influenced by varying the methylation levels [34,35]. It has been shown that human mtDNA have both CpG and Non-CpG methylation sites [36,37] and mitochondrial genome methylation patterns can vary between normal and cancer cells [29]. 5-aza-dC is a DNA methyltransferase inhibitor that can be used to detect the methylation status of tissues and how mtDNA variations alter the methylation profile of different genes. Nashine et al. found that mitochondria from AMD subjects can affect epigenetic modulation in transmitochondrial cybrids [38,39]. Additionally, cells with either H or J haplogroup mtDNA were shown to have different levels of total global methylation and expression of methylation-related nuclear genes [40,41].

Gene expression can be regulated by how tightly DNA associates with the nuclear proteins called histones, which are responsible for nucleosome structure integrity and gene regulation in eukaryotes. It has been shown that certain racial/ethnic populations are more prone to develop specific diseases [42,43,44,45,46,47] and also that epigenetics play a role in disease progression.

Our cybrid results demonstrate that the mitochondria with African L haplogroup mtDNA can differentially regulate epigenetic-related pathways compared to European H mtDNA mitochondria. In summary, the maternal mtDNA from the African versus European populations can contribute to different epigenetic profiles that in turn may differentially up- or downregulate specific major pathways critical for disease processes. Understanding these differences may help us identify targets for future therapeutics.

## 2. Materials and Methods

### 2.1. Cybrid Cultures

Institutional review board (IRB HS#2003-3131) approval was obtained from the University of California-Irvine. Tubes containing sodium citrate buffer were used to collect 20 mL of peripheral blood by venipuncture for DNA analyses. DNA was isolated from blood with a DNA extraction kit (PUREGENE, Qiagen, Valencia, CA, USA). A series of centrifugation steps were performed to isolate platelets from the blood samples. Final pellets were suspended in Tris-buffered saline. Cultures of ARPE-19 cells were made deficient in mtDNA (Rho^0^) by serial passage in low-dose ethidium bromide. Cybrids were created by polyethylene glycol fusion of platelets with Rho^0^ ARPE-19 cells according to modified procedures of Chomyn [48]. Cybrids were cultured until confluent in DMEM-F12 containing 10% dialyzed fetal bovine serum, 100 unit/mL penicillin, 100 μg/mL streptomycin, 2.5 μg/mL fungizone, 50 μg/mL gentamycin, and 17.5 mM glucose. Cybrid haplogroup profiles were confirmed by matching the cybrid haplogroup with the subject haplogroup from the blood DNA. For each experiment, all H and L cybrids were at passage 5 and all experiments were performed under identical controlled conditions.

### 2.2. Identification of Cybrid Haplogroups

Cybrid DNA was extracted from cell pellets using a spin column kit (DNeasy Blood and Tissue Kit, Qiagen, Valencia, CA, USA) as described previously [31,49]. Mitochondrial haplogroups were identified by PCR along with restriction enzyme digestions, allelic discrimination, and sequencing [31,40,49]. The major defining SNP for the H haplogroup is T7028C. The major SNP defining the L haplogroup is C3594T. The mtDNA sequences were compared with classification from www.phylotree.org and www.MitoMap.com (accessed on 16 July 2020) The entire mtDNA sequences for H cybrids (n = 5) were categorized as H1b5, H66a, H4a1a4b2, H4a1a4b, H11a2a2, H1, and H1j. The L haplogroup cybrids (n = 5) were categorized as L0a1a1, L1b2a, L2b2, L1b2a, L1c2a1, and L1b1a7. The rest of the details are mentioned in Supplementary Table S2.

### 2.3. Amyloid-β Treatment of H versus L Cybrids

#### 2.3.1. Amyloid-β Treatment of H versus L Cybrids

The amyloid-β_1-42_ (active form) and amyloid-β_42-1_ (inactive form) peptides were reconstituted according to the manufacturer’s (AnaSpec, Fremont, CA, USA) directions by diluting the initial material in 1% NH_4_OH to a 2.77 mM stock solution. This was further diluted in 1 × PBS to 100 µM, which was stored in aliquots at −20 °C. At the time of use, aliquots were thawed and diluted to 20 µM Amyβ with media. The H (n = 5) and L (n = 5) cybrids were initially plated for 24 h and then fresh media plus 20 µM Amyβ and the untreated samples had their culture media changed.

#### 2.3.2. Mitochondrial Membrane Potential (ΔΨM) Assay

The H (n = 5) and L (n = 5) cybrids were plated at 10,000 cells/well in 96-well plates and incubated for 24 h. Cells were cultured for 24 h in the presence of 20 µM Amyβ. The JC-1 reagent (5,5′,6,6′-tetrachloro-1,1′,3,3′-tetraethylbenzimidazolylcarbocyanine iodide) was used according to the manufacturer’s (Biotium, Hayward, CA, USA) instructions. After a 15 min JC-1 treatment period, fluorescence was measured using red (excitation/550 nm and emission/600 nm) and green (excitation/485 nm and emission/545 mm) wavelengths (Gemini XPS Microplate Reader, Molecular Devices, San Jose, CA, USA). Intact mitochondria with normal ΔΨM appeared red, while cells with decreased ΔΨM were in a green, fluorescent state. Experiments were analyzed in quadruplicate and the entire experiment was repeated three separate times. The Amyβ treated values were compared to untreated values for statistical significance (*p* ≤ 0.05, GraphPad Prism Software, Inc., San Diego, CA, USA).

#### 2.3.3. Reactive Oxygen Species (ROS) Assay

The H (n = 5) and L (n = 5) cybrids were seeded at 10,000 cells/well in 96-well plates and incubated for 24 h. The fluorescent H_2_DCFDA dye (2,7-dichlorodihydrofluorescin diacetate, Invitrogen-Molecular Probes, Carlsbad, CA, USA) was added to each well and measured using a fluorescence plate reader (490 nm for emission and 520 nm for excitation wavelengths, Gemini XPS Microplate Reader, Molecular Devices, Sunnyvale, CA, USA). Results were normalized to the untreated H cybrid or L cybrid using Prism-GraphPad Software (San Diego, CA, USA) and were statistically significant when *p* ≤ 0.05. Each condition had quadruplicated replicates and the experiments were repeated 3 times.

#### 2.3.4. Cell Metabolism Assay

To measure the cellular metabolism, H (n = 5) and L (n = 5) cybrids were plated at 10,000 cells per well and cultured for 24 h prior to being treated with amyloid-β_1-42_. After being cultured for an additional 24 h, the 10 µL MTT reagent (3-(4,5-Dimethylthiazol-2-yl)-2,5-diphenyltetrazolium bromide, Biotium, Hayward, CA, USA) was added for 2 h to each well, after which the reaction was quenched with 100 µL DMSO. The samples were read using an absorbance reader at 570 nm (MTT) and 630 nm (background). The values were normalized to the corresponding untreated values. Each condition was run in six replicates, and the experiments were repeated three times.

### 2.4. Sub-Lethal UV Radiation Treatment of H versus L Cybrids

#### 2.4.1. Growth Curve Assay after UV Exposure

The H and L cybrids were plated at 300,000 cells per well in 6-well plates and cultured for 24 h. The media were removed and the cybrids were then treated with a single 10 s pulse at 10 mJ UV radiation (Stratalinker 1800, Agilent, La Jolla, CA, USA). The media were replaced, changed every 48 h, and the growth curve was measured using a trypan blue dye exclusion assay along with the Cell Viability Analyzer (Vi-CELL XR, Beckman Coulter, Miami, FL, USA). This assay uses 50 images to count cells and values are averaged. The 0 time point was designated as 100%, and the percentage increase was determined at 48, 96, 120, and 144 h. The trypan blue assay was run with biological triplicates and each experiment was repeated twice.

#### 2.4.2. Quantitative Real-Time PCR (qRT-PCR) Analyses

The RNA was isolated from the UV-treated and untreated European-H (n = 5) and African-L (n = 5) cybrids at 0, 72, and 120 h after UV exposure with the RNeasy Mini-Extraction kit (Qiagen, Inc.) following the manufacturer’s protocol. After being quantified (NanoDrop 1000, ThermoFisher Scientific, Inc., Waltham, MA, USA) the RNA was reverse transcribed into cDNA with the QuantiTect Reverse Transcription Kit. The details for the following genes used in the UV radiation studies can be found in Supplementary Table S3: Complement Factor H (*CFH*), *CD55*, *CD59*, Interleukin-33 (*IL33*), Transforming Growth Factor alpha (*TGFA*), EGF-containing fibulin-like extracellular matrix protein 1 (*EFEMP1*), Retinoic Acid Receptor alpha (*RARA*), BCL binding component 3 (*BBC3*), and BCL2-like 13 (*BCL2L13*). qRT-PCR was performed using the QuantiFast SYBR Green PCR kit on a Bio-Rad iQ5 iCycler detection system. The Hypoxanthine-guanine phosphoribosyl transferase (HPRT1, NM-000194) was used as a reference gene. Samples were assayed in triplicate or quadruplicate. The delta Ct was calculated as the difference between the Ct (threshold cycle) of the target gene and the Ct of the housekeeper/reference gene. The delta-delta Ct was calculated as the mean difference of delta Cts of a specific gene at a single time point between the H and L cybrids or delta Cts of the genes being compared at two different time points of either the H or L cybrids. The fold values were calculated using the formula Fold = 2^ΔΔCT^. Fold values were calculated relative to the readings for corresponding H cybrids at 0 h. Data are presented as mean ± standard error of the mean (SEM). Experiments were performed in triplicate. *p*-values < 0.05 (two-tail test) were considered statistically significant.

### 2.5. Study of Epigenetics of H versus L Cybrids

#### 2.5.1. Inhibition of Methylation in Cybrid Cultures

These experiments were designed to determine if the European-H versus African-L mtDNA cybrids responded differently to inhibition of methylation in their RNA expression levels for downstream nuclear genes. H cybrids (n = 5) and L cybrids (n = 5) were plated for 24 h, then media were removed and replaced with the same media containing a final concentration of 250 μM 5-Aza-2′ deoxycytidine (5-aza-dC), an inhibitor of methylation (Sigma-Aldrich, St. Louis, MO, USA), for 48 h treatment. Culture media were replaced after each 24 h period with fresh media containing the compound. Cells were pelleted, RNA isolated, and cDNA synthesized as described below.

#### 2.5.2. Global DNA Methylation Assay of Cybrid Cultures

The global DNA methylation status was detected using the MethylFlash Methylated DNA Quantification Kit (EpiGenTek, Farmingdale, NY, USA) according to the manufacturer’s protocol. The amount of DNA used in the assay was 110 ng. Briefly, the H (n = 5) and L (n = 5) cybrids were cultured until confluent and DNA isolated as described earlier. The DNA was bound to strip wells that have a high DNA affinity. The methylated fraction of DNA was detected using capture and detection antibodies and then quantified with an ELISA-like reaction in a microplate spectrophotometer (absorbance 450 nm). The amount of methylated DNA (5-mC%) was proportional to the OD intensity measured with an absorbance plate reader (Bio-Tek, Winooski, VT, USA), calculated according to the kit’s formulas for the relative methylation status of two different DNAs. Samples were run in duplicate, and the experiment was repeated.

#### 2.5.3. Gene Chip Expression Assay and Statistical Analyses

For the gene expression analyses, the RNAs from the three H haplogroup cybrid cultures were combined (250 ng/mL per sample) into a single sample for analyses. Three L haplogroup cybrid cultures were also combined into one sample. The H cybrid and L cybrid RNAs were sent to the UCLA Clinical MicroArray Core Lab for analyses with the Affymetrix Human U133 Plus 2.0 Array. The gene expression results were analyzed with pathway analysis software (INGENUITY Systems, Redwood City, CA, USA).

#### 2.5.4. Quantitative Real-Time PCR (qRT-PCR) Analyses

Cells from cybrid cultures were pelleted and RNA isolated using the RNeasy Mini Extraction kit (Qiagen) as described previously. This study compares relative differences in gene expression between the H and L cybrids. For qRT-PCR analyses, 100 ng of individual RNA samples were reverse transcribed into cDNA using a QuantiTect Reverse Transcription Kit (Qiagen, Inc.). qRT-PCR was performed in triplicate using 14 different primers (QuantiTect Primer Assay, Qiagen, Inc.) for genes associated with various aspects of acetylation (*HAT1*, *HDAC1*, *HDAC6*, *HDAC11*, and *SIN3A*), methylation (*MAT2B*, *MBD4*, *DNMT1*, *DNMT3A*, and *DNMT3B*), histone proteins (*HIST1H3A*, *HIST1H3F*, *HIST1H3H*, and *HIST1H4H*), ubiquitination (*USP2*, *USP3D*, *USP25*, *USP34*, and *USP53*), signaling and angiogenesis pathways (*NFKB1*, *NFKBIA*, *IL33*, *TRADD*, *TRAF1*, and *VEGFA*), and chemokines (*CXCL1*, *CXCL5*, and *CXCL8*). The qRT-PCR was performed on individual samples using a QuantiFast SYBR Green PCR Kit (Qiagen) on a Bio-Rad iCycler iQ 500 detection system. For the various target genes, housekeeping genes that had comparable amplification efficiencies to the genes of interest were chosen in order to maximize the accuracy of our ΔΔCT values. The housekeeping genes were either *HPRT1*, *HMBS*, *ALAS1*, or *TUBB*.

### 2.6. Statistical Analyses

Statistical analyses of the data were performed by ANOVA (GraphPad Prism, version 5.0 and version 9.3). Newman–Keuls and sidak multiple comparison or two-tailed *t*-tests were used to compare the data within each experiment.

## 3. Results

### 3.1. Comparison of the Effects of Amyloid Treatment on H and L Cybrids

**Figure 1:**
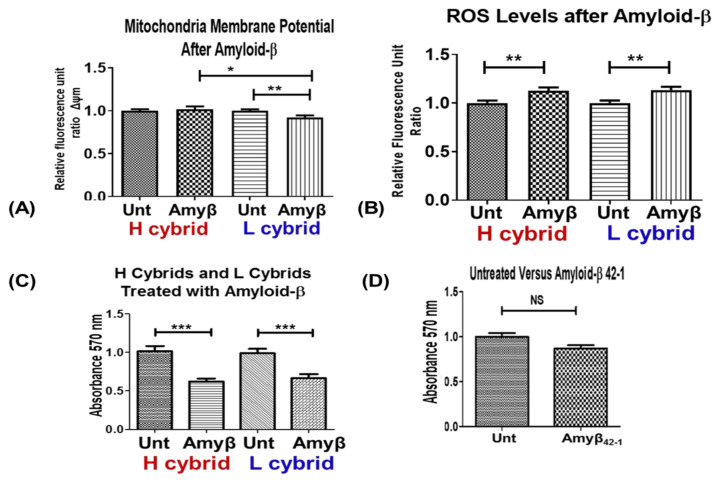
**Exploring the difference in responsiveness to Amyloid-**β **treatment between H (European) and L (African) cybrids.** (**A**) The effect of Amyloid-β treatment on the mitochondrial membrane potential (ΔψM) of H and L cybrids. H (n = 5) and L (n = 5) cybrids were grown for 24 h in the presence of 20 M Amyβ. The JC-1 assay was used to assess ΔψM. (**B**) Amyloid-β treatment had an effect on the reactive oxygen species (ROS) of H (n = 4) and L (n = 5) cybrids. The same conditions as above were used to measure the ROS using H2DCFDA dye. (**C**) The MTT assay was used to determine cellular metabolism. Amyβ was applied to H and L cybrids after they had been plated and grown for 24 h. (**D**) Cybrids were exposed to the scrambled Amyβ_42-1_ peptides (inactive form) and cellular metabolism measured using MTT assay * *p*-value ≤ 0.05, ** ≤ 0.01, *** ≤ 0.001 and NS: non-significant.

The H and L cybrids were cultured in the presence of amyloid-β_1-42_ peptide (active form) and analyzed for changes in levels of ΔΨM, ROS, and metabolism. The 20 µM-Amyβ-treated L cybrids showed a 7.9% decline in ΔΨM compared to untreated L cybrids (0.92 ± 0.025, n = 5 versus 1.00 ± 0.015, n = 5, *p* = 0.009), while the H cybrids showed no significant change (1.00 ± 0.08 versus 1.014 ± 0.034, *p* = 0.7) (Figure 1A). The ΔΨM in the treated L cybrids were significantly lower than the treated H cybrids (1.01 ± 0.035 versus 0.93 ± 0.025, *p* = 0.03). After treatment with Amyβ, the ROS levels increased in H cybrids (1.00 ± 0.025 versus 1.13 ± 0.03, *p* = 0.004) and L cybrids (1.00 ± 0.27 versus 1.14 ± 0.02, *p* = 0.002) (Figure 1B). The cell metabolism declined 38.1% in the Amyβ-treated H cybrids compared to untreated H cybrids (1.01 ± 0.07 versus 0.62 ± 0.042, *p* = 0.0001). The treated L cybrids also showed a decrease in metabolism after Amyβ exposure (0.99 ± 0.058 versus 0.67 ± 0.053, *p* < 0.001) (Figure 1C). As a control, cybrids were exposed to the scrambled Amyβ_42-1_ peptides (inactive form) and showed similar cell metabolism levels (0.87 ± 0.034) compared to untreated cybrids (1.00 ± 0.18, *p* = 0.2) (Figure 1D). Our findings suggest that after Amyβ_1-42_ treatment, the European-H and African-L cybrids responded differently with respect to ΔΨM levels but had similar changes in ROS and cell metabolism levels.

### 3.2. Sub-Lethal UV Radiation Treatment of H versus L Cybrids

#### 3.2.1. Comparison of Growth Curves in H and L Cybrids after UV Treatment

**Figure 2:**
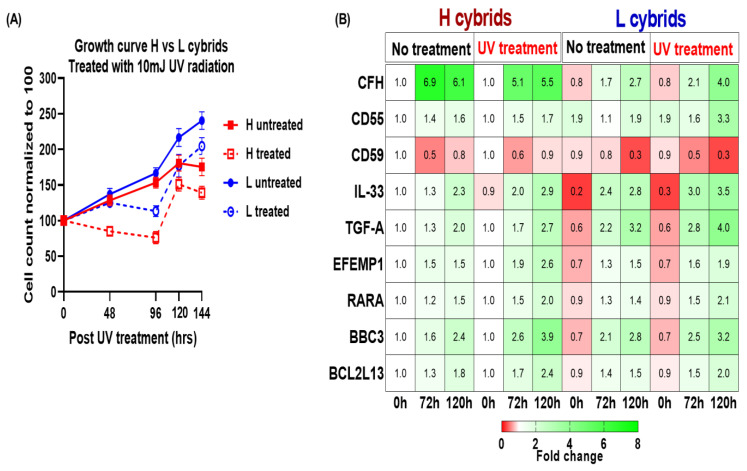
**Evaluating the H (European) and L (African) hybrids’ responses to UV radiation treatment.** (**A**) In both UV-treated and untreated groups, hybrids having L haplogroup mtDNA had a steeper growth curve slope than H haplogroup mtDNA. Untreated, both H and L cybrids showed consistent increases in growth patterns. Growth rates in UV-treated samples slowed or declined until about 96 h after treatment, then increased again. The trypan blue assay was performed with technical and biological replicates, and each experiment was repeated twice. The red solid line represents untreated H cybrids; the red broken line represents UV-treated H cybrids; the blue solid line represents untreated L cybrids; and the blue broken line represents UV-treated L cybrids. (**B**) Analysis of the differential expression of CFH, CD55, CD59, IL-33, TGF-A, EFEMP1, RARA, BBC3, and BCL2L13 in H and L cybrids exposed to UV light for 0, 72, and 120 h with and without treatment. Heatmap representation of CFH, *CD55*, *CD59*, *IL-33*, *TGF-A*, *EFEMP1*, *RARA*, *BBC3*, and *BCL2L13* differential expression. The numbers in the heatmap show the average relative fold-change values.

At the 0 h time point, the cell counts were normalized to 100% for both the untreated (Control) and UV-treated (Experimental) groups (Figure 2A). The growth curves for the untreated European-H and African-L cybrids showed similar increases until 120 h, when the L cybrids showed a 30% increase compared to the H cybrids (222% versus 192%). At 144 h, the untreated L cybrids had increased to 256%, compared to the untreated H cybrids (199%).

After UV treatment, both L and H cybrids showed an initial decline in growth compared to the untreated cybrids. At 96 h, the growth rate of the UV-treated H cybrids decreased to 72%, while the UV-treated L cybrids had increased to 115%. The UV-treated L cybrids showed higher growth rates than UV-treated H cybrids at 120 h (185% versus 156%) and 144 h (209% versus 148%).

#### 3.2.2. Comparison of Gene Expression Profiles in H and L Cybrids after UV Treatment

Next, we wanted to test the effect of UV treatment on the genes representing pathogenic pathways in eye illnesses such as AMD and diabetic retinopathy and how they differed in European H cybrids from African L cybrids. The complement pathway (CFH, CD55, CD59) has been linked to the development and severity of AMD. Apoptosis is also a characteristic hallmark of retinal diseases, so BBC3 and BCL2L13, RARA were investigated, as well as genes (EFEMP1, CFH) involved in the regulation of methylation sites. The values of untreated H cybrids at 0 h were normalized to 1, and the expression levels of the nine genes (Supplementary Table S3) for the European-H and African-L cybrids at 72 and 120 h are presented as Mean ± SEM (Figure 2B, Supplementary Table S4). ***CFH*:** At 72 h, the untreated H cybrids had significantly higher *CFH* levels compared to untreated L cybrids (6.9-fold ± 0.15 versus 1.68-fold ± 0.12; SE of difference = 0.19, *p* < 0.0001). The higher levels were also found at 120 h with untreated H cybrids and L cybrids (6.11-fold ± 0.12 versus 2.72-fold ± 0.078, SE of difference = 0.14, *p* < 0.0001). After UV treatment, the H cybrids showed significantly higher *CFH* levels at 72 h (5.14-fold ± 0.11 versus 2.09-fold ± 0.10, SE of difference = 0.15, *p* < 0.0001) and 120 h (5.52-fold ± 0.12 versus 3.99-fold ± 0.15, SE of difference = 0.20, *p* < 0.0001) compared to UV-treated L cybrids. ***IL33*:** At 0 h, the IL33 levels for untreated H cybrids were higher than the untreated L cybrids (1-fold ± 0 versus 0.23-fold ± 0.02, *p* < 0.0001). After UV treatment, the H cybrids showed a significant decrease in *IL33* at 72 h compared to UV-treated L cybrids (1.98 ± 0.14 versus 2.94 ± 0.04, *p* = 0.0002) and 120 h (2.88 ± 0.04 versus 3.45 ± 0.09, *p* = 0.0002). Moreover, at 120 h, the UV-treated H cybrids showed significantly elevated *IL33* compared to untreated H cybrids (2.97 ± 0.15 versus 2.08 ± 0.13, *p* = 0.001), while the *IL33* expression levels in untreated and UV-treated L cybrids were similar (*p* = 0.06). ***EFEMP1*:** At 0 h the untreated L cybrids expressed lower levels of *EFEMP1* than untreated H cybrids (0.69 ± 0.05 versus 1 ± 0.0, *p* < 0.0001). By 120 h, the UV-treated H cybrids showed higher expression levels of *EFEMP1* (2.57 ± 0.05) compared to the UV-treated L cybrids (1.94 ± 0.02, *p* < 0.0001). ***BBC3*:** At 120 h, the UV treatment significantly increased expression levels of *BBC3* compared to the untreated controls; (UV-H cybrid, 3.90 ± 0.09 versus untreated H, 2.37 ± 0.04, *p* < 0.0001) and (UV-L cybrid, 2.96 ± 0.09 versus untreated L cybrid, 2.79 ± 0.05, *p* = 0.0003). There were no significant differences in expression levels between the H cybrids and L cybrids after UV treatment for *CD55*, *CD59*, *TGFA*, *RARA*, and *BCL2L13*.

In summary, our results demonstrate that, 120 h after sub-lethal UV radiation, the European-H cybrids showed significantly higher expression levels of complement/inflammation genes (*CFH*, *IL33*, and *EFEMP1*) compared to the UV-treated African-L cybrids Section 3.2.1.

### 3.3. Study of Epigenetics of H versus L Cybrids

#### 3.3.1. Elevated Levels of Global DNA Methylation in L versus H Cybrids

**Figure 3:**
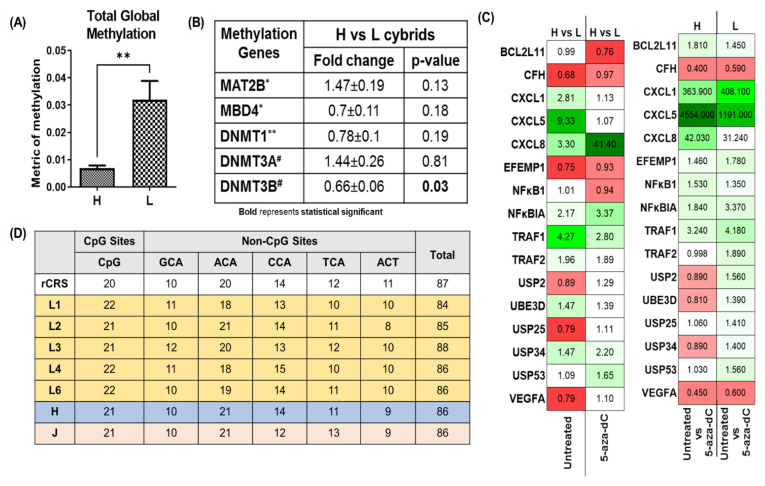
**Evaluating the status of methylation in cybrids including mitochondrial haplogroups of European (H) and African (L) ancestry**. (**A**) African-L cybrids showed increased levels of total global methylation. H (n = 5) and L (n = 5) cybrids were grown under identical conditions and DNA was extracted. The methylation fraction of DNA was detected using capture and detection antibodies and measured in a microplate spectrophotometer using an ELISA-like reaction (absorbance 450 nm). The OD intensity measured using an absorbance plate reader was proportional to the amount of methylated DNA (5-mC%). The L cybrids had a higher 5-mC% mean value compared to the H cybrids (*p* = 0.005). To produce biological duplicates, samples were run in duplicate, and the experiment was repeated twice. (**B**) Expression levels of methylation genes in H cybrids versus L cybrids. Fold-change values greater than 1 indicate upregulation of the gene and those less than 1 indicate down-regulation of the gene compared to H cybrids. H cybrids are assigned a value of 1. * n = 5 different H cybrids and n = 5 different L cybrids, with three values for each sample, ** n = 5 different H cybrids and n = 5 different L cybrids, with three values for each sample, ^#^ n = 5 different H cybrids and n = 5 different L cybrids, with three values for each sample. (**C**) Heatmap representation of the differential expression of *BCL2L11*, *CFH*, *CXCL1*, *CXCL5*, *CXCL8*, *EFEMP1*, *NFκB1*, *NFκBIA*, *TRAF1*, *TRAF2*, *USP2*, *UBE3D*, *USP25*, *USP34*, *USP53*, and *VEGFA* genes before and after treatment with 5-aza-dC in H and L cybrids. (**D**) Potential methylation sites in the MT-Dloop (16411-460) for H and L cybrids compared to revised Cambridge Standard (rCRS). H and J represent cybrids created from 5 different individuals [40].

All cybrid cultures were grown under identical conditions and for identical periods of time. The results showed that the 5-mC% mean value for the European-H hybrids was 0.007 ± 0.001 and the metric of methylation mean value for the African-L cybrids was 0.032 ± 0.007 (Figure 3A). The mean difference for the H versus the L cybrids was −0.0251 ± 0.007 (*p* = 0.005). This indicates that the L cybrids showed significantly higher levels of total global methylation compared with the H cybrids.

#### 3.3.2. Altered Expression Levels of Methylation Genes

The expression levels for five genes related to methylation (Supplementary Table S6) were analyzed by qRT-PCR in European-H cybrids versus African-L cybrids. In these studies, the H cybrids were assigned a value of 1, and the L cybrid expression levels were either up-regulated (value greater than 1) or down-regulated (value less than 1). The expression level for the *DNMT3B* gene, which methylates de novo during development, was lower in the L cybrids compared with the H cybrids (0.66-fold ± 0.06, *p* = 0.03) (Figure 3B). The values for H cybrids and L cybrids were similar for *MAT2B*, *MBD4*, *DNMT1*, and *DNMT3A*.

#### 3.3.3. Methylation Inhibitor Studies Comparing African-L versus European-H Cybrids

The H and L cybrids were treated with 5-aza-dC, a methylation inhibitor, and 16 genes (Supplementary Table S3) related to complement, angiogenesis, inflammation, signaling, and ubiquitination were measured before and after demethylation. The *CFH* expression levels in the untreated L cybrids and untreated H cybrids were similar (*p* = 0.17) but, after 5-aza-dC treatment, the expression levels for *CFH* in the treated H cultures decreased significantly (0.4 ± 0.03-fold, *p* = 0.018), while those for the L-treated did not change (*p* = 0.11) (Figure 3C, Supplementary Table S6). The *EFEMP1* gene expression levels were decreased in the untreated L cybrids compared to untreated H cybrids (0.75 ± 0.07-fold, *p* = 0.04). After treatment with 5-aza-dC, the L-treated cells expressed significantly higher *EFEMP1* compared to the L-untreated (1.78 ± 0.22-fold, *p* = 0.033), while the H-treated expression was similar to that of the H-untreated (*p* = 0.13).

The *VEGFA* gene was expressed at similar levels in the untreated L cybrids and the untreated H cybrids (0.79 ± 0.07-fold, *p* = 0.12, Figure 3C, Supplementary Table S6). However, after the 5-aza-dC treatment, the H-treated and L-treated cybrids showed significantly lower expression levels (0.45 ± 0.04, *p* = 0.003 and 0.64 ± 0.06, *p* = 0.029, respectively) compared to the untreated cells. This indicates that demethylation downregulated the expression of *VEGFA*. The 5-aza-dC treatment also significantly upregulated *CXCL1* and *CXCL8* in the H-treated cybrids (363.9 ± 25.84, *p* = 0.0002 and 42.03 ± 11.9, *p* = 0.03, respectively). The L-treated cybrids showed trends of upregulation but the variability between samples lead to non-significance. Finally, the treated L cybrids showed increased expression of *USP25*, a ubiquitin-specific peptidase, that mediates disassembly of the polyubiquitin chains (1.41 ± 0.13, *p* = 0.04).

#### 3.3.4. Sequence Comparisons of H and L Cybrids for the MT-Dloop

Using cybrids L (n = 5), H (n = 5), and J (n = 5) the MT-Dloop control region from nucleotides 16411-460 was sequenced and analyzed (Figure 3D). Potential methylation sites, grouped into CpG or Non-CpG sites, were counted and compared to the Cambridge Reference Sequence (rCRS). Non-CpG sites are represented by the sequences GCA, ACA, CCA, TCA, and ACT. In the present study, the L cybrids showed slight variations in numbers of CpG sites (range 21 to 22) compared to the H (21 sites) and J cybrids (21 sites). In the Non-CpG category, the L cybrids showed a range of 10 to 12 GCA sites, while the rCRS H cybrids and J cybrids had 10 GCA sites. The L cybrids had a range of ACA sites (18 to 21) while the H and J cybrids had 21 sites. The CCA sites ranged between 13 to 15 in L cybrids compared to the H cybrids (14 sites), J cybrids (12 sites), or the rCRS (14 sites). The TCA sites showed slightly lower numbers in L cybrids (10 to 12) compared to the J cybrids (13 sites). The total numbers of methylation sites within the L cybrids varied from 84 to 86 sites compared to the H sites (86), J sites (86), and rCRS (87 sites).

### 3.4. Acetylation Studies

#### 3.4.1. Expression of Acetylation Genes

**Figure 4:**
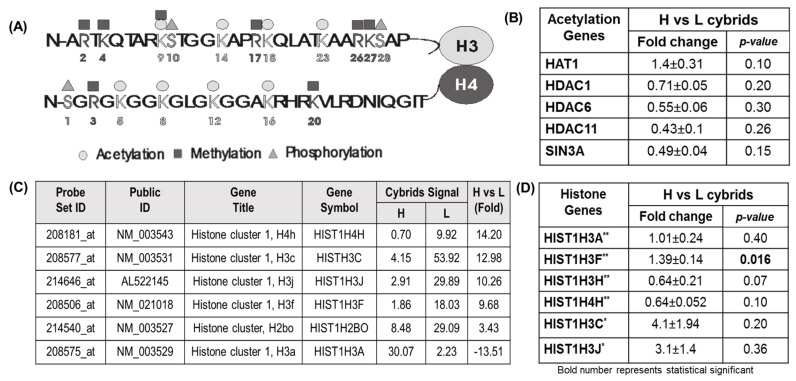
**Assessing the acetylation status of cybrids from European (H) and African (L) ancestry.** (**A**) A schematic representation of the sites where epigenetic modification occurs in the N-terminus portions of the histones H3 and H4. (**B**) Acetylation gene expression levels in H cybrids versus L cybrids. When compared to H cybrids, fold-change values larger than 1 indicate gene upregulation and those less than 1 indicate gene downregulation. The value 1 is assigned to H cybrids. (**C**) Affymetrix Chip data comparing H cybrids versus L cybrids for histone genes. (**D**) Histone gene expression levels in H cybrids versus L cybrids. As mentioned above, the fold-change value representation is employed. European-H cybrids (* n = 5) and African-L cybrids (n = 5), with three values for each sample, European-H cybrids (** n = 5) and different African-L cybrids (n = 5), with three values for each sample.

Five genes associated with acetylation were examined in the European-H and African-L cybrids. The functions included the addition of acetyl groups (*HAT1*) and deacetylation (*HDAC1*, *HDAC6*, *HDAC11*, and *SIN3A*) (Supplementary Table S7, Figure 4A). The qRT-PCR results showed that the expression levels for *HAT1*, *HDAC1*, *HDAC6*, *HDAC11*, and *SIN3A* were similar in the H and L cybrids (Figure 4B).

#### 3.4.2. Differential Expression Levels for Histone Cluster Genes in L versus H Cybrids

Based upon differences found between H cybrids and L cybrids in the growth studies as well as the energy pathway assays [31,40], we hypothesized that the H and L cybrids would have different expression patterns of the histone protein genes. The Affymetrix Chip data suggested higher fold levels would be found in the L cybrids compared to H cybrids (Figure 4C). However, when qRT-PCR for the cluster histones (Supplementary Table S8) were performed on H (n = 5) and L (n = 5) cybrids, only the *HIST1H3F* histone showed a significant increase in L cybrids compared to the H cybrids (1.39 ± 0.14, *p* = 0.016) (Figure 4D). The other histone genes were expressed at comparable levels in H and L cybrids.

## 4. Discussion

*Response to Stressor Studies:* Two different stressors were used in the present study, Amyβ_1-42_ and sub-lethal UV radiation. The accumulation of Amyβ, along with mitochondrial damage and dysfunction, has been reported in AMD and Alzheimer’s disease [15,21,27,50]. The Amyβ_1-42_ treatment resulted in a significant decrease in the mitochondrial membrane potential (ΔΨm) for the African-L cybrids while the ΔΨm levels for Amyβ-treated European-H cybrids were not changed. The ΔΨm represents a proton gradient generated by Complexes, I, III, and IV of the electron transport chain. The decrease in the ΔΨm, as measured by the JC1 assay, can represent the earliest events that can lead to apoptosis. As the ΔΨm collapses, the cascade to apoptosis becomes irreversible. Our findings that the L cybrids show lower ΔΨm levels when treated with Amyβ_1-42_ suggest that the maternal African mitochondria may be more susceptible and less stable than the European mitochondria. Since Amyβ_1-42_ is a common stressor in aging diseases, this difference in African versus European mitochondrial sensitivity may play a role in different incidence of these Amyβ_1-42_-related diseases.

Both H and L cybrids showed increased ROS and lower cell metabolism in response to Amyβ_1-42_ treatment. The decline in cell metabolism after exposure to Amyβ_1-42_ was also shown in cybrids containing the K mtDNA haplogroup, which is associated with the Ashkenazi Jewish population [30]. Since all of the RPE cybrid cell lines have identical nuclear genomes, our data support the retrograde signaling concept that specific mtDNA haplogroup representing diverse populations can influence the cellular responses to Amyβ, a toxic protein associated with aging diseases.

**Figure 5:**
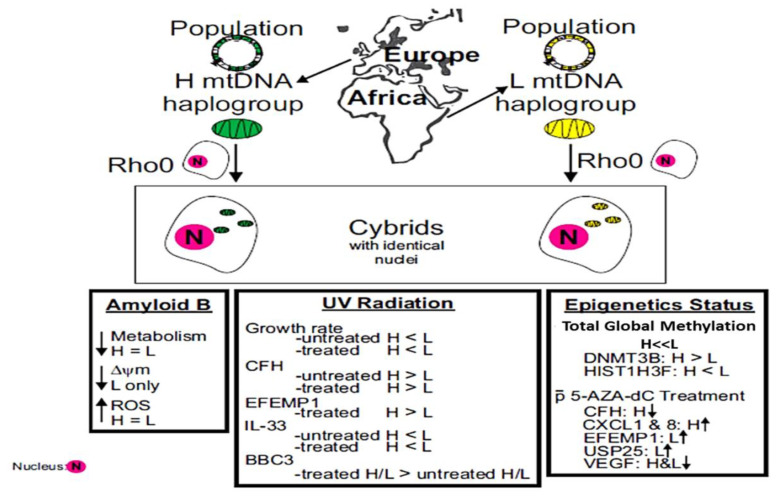
A schematic illustration of how the maternal mtDNA from African populations versus European populations can lead to various epigenetic profiles, which in turn may differentially regulate specific key pathways that are crucial for disease processes.

Due to a lack of introns and poor repair processes, exposure to UV radiation generates 10-fold higher levels of mutations within the mtDNA compared to nuclear DNA. In the untreated and UV-treated European-H cybrids, the expression levels for *CFH* and *BBC3* were significantly higher than those found in the African-L cybrids. CFH is an important inhibitor for the alternative complement pathway, provides protection against complement activation, and may be associated with the H mtDNA haplogroup being at lower risk for AMD compared to other European mtDNA haplogroups [51]. Recently, Ebeling et al. used iPSC-RPE cells to show that CFH high-risk cells had decreased mitochondrial functions along with higher inflammatory markers [51]. IL33, a cytokine in the IL-1 superfamily, is a major regulator for inflammatory cytokines and retinal photoreceptor degeneration. This cytokine also blocks ocular angiogenesis and modulates tissue remodeling. IL33 serves as a metabolic checkpoint that stabilizes RPE cells, regulates mitochondrial metabolism and oxidative pyruvate catabolism, and influences mitochondrial morphology. The UV-treated H cybrid had over 2-fold higher expression of *EFEMP1* compared to the UV-treated L cybrids. *EFEMP1* is a high-risk gene for AMD and is expressed in RPE cells where it becomes misfolded and accumulates within drusen. Furthermore, EFEMP1 accumulation has been reported as a biomarker for choroidal neovascularization in AMD. Since the European population is more likely to develop AMD than African populations, perhaps exposure to UV might elevate *EFEMP1* and *IL33* levels, which may contribute to the early retinal changes (Figure 5).

*Total Global Methylation Studies:* Recent studies have shown variabilities in total global methylation levels in individuals and human cybrids with different European mtDNA haplogroups [30,40] as well as mtDNA methylation correlating positively with SNP density and age and negatively with mtDNA gene expression [50]. Moreover, mitochondrial methylation patterns have been shown to vary between normal and cancer cell lines [29].

Our study illustrates that cybrids containing the African-L haplogroup mtDNA have significantly higher levels of total global methylation compared with European-H cybrids (*p* = 0.005). Thaker et al. also reports that European-H cybrids also have lower total global methylation levels than the more bioenergetically active K cybrids (*p* = 0.013) [30]. Since total methylation varied in European-H versus African-L cybrids, the expression levels of five methylation-specific genes were also examined. We showed that the expression level for the *DNMT3B* gene was lower in African-L cybrids compared to the European-H cybrids (*p* = 0.03), which was a similar pattern to that seen in the European-J cybrids (*p* < 0.001) [40].

Patil et al. showed that DNMT3B knockdown cells displayed a comparatively pronounced global reduction in mtDNA methylation with concomitant increases in gene expression, suggesting a potential functional link between methylation and gene expression [29]. The European-H cybrids and African-L cybrids had similar expression patterns for the *MAT2B*, *MBD4*, *DNMT1*, and *DNMT3A* genes. This contrasts with the European-J cybrids, which preferentially use glycolysis and showed elevated *MAT2B* (*p* = 0.002) but lower expression of *DNMT1* and *DNMT3A* (*p* < 0.0001) compared to the European-H cybrids [40]. The varied levels of *DNMT3B* (Figure 5) in the African-L cybrids compared to the cybrids with the European-H haplogroup may play a role in the differential susceptibility of African Americans to various diseases compared to Caucasian Americans. Further studies will be needed to investigate this topic.

The *DNMT* genes encode for proteins that preferentially methylate hemimethylated DNA at CpG residues during the DNA replication S phase to ensure epigenetic inheritance. *DNMT1* has been identified inside mitochondria while *DNMT3A* has recently been associated with mitochondrial fractions [52]. Bellizzi and coworkers showed that DNA methylation occurs in the mtDNA control region of mammals and that inactivation of the *DNMT1*, *DNMT3A*, and *DNMT3B* genes in mouse embryonic stem cells resulted in a reduction in CpG methylation and had no effect on Non-CpG methylation [36]. In humans, the NGS-bisulfite sequencing technique showed that the mitochondrial genome is heavily methylated with predominantly non-CpG methylation and concomitant expression of varying DNMT enzymes. Furthermore, Dou et al. showed that *DNMT3A* modulates mtDNA methylation, especially between *MT-ND2* and *MT-CO1* [50]. It is known that DNA methylation levels can provide valuable insights into abnormalities of mitochondrial metabolism; however, further studies are needed to understand the mechanisms by which the methylation status of mtDNA affects the incidence of diseases.

*Demethylation Studies Using 5-aza-dC*: Having shown that European-H and African-L cybrids have different levels for total global methylation and expression of methylation genes, we wanted to investigate the influence of European-H versus African-L mtDNA upon nuclear genes known to be regulated by methylation. The development of AMD is associated with *CFH* (an inhibitor for alternative complement), *EFEMP1* (a high-risk gene for AMD and other retinal degenerations), and *VEGFA* (a protein associated with increased choroidal neovascularization) [53,54,55,56,57]. In addition, inflammation is a hallmark event in AMD. Therefore, we also examined genes known to have pro-inflammatory effects (e.g., *IL33*, *CXCL1*, *CXCL5*, and *CXCL8*).

The H and L cybrids were cultured with and without 5-aza-dC, a DNA methyltransferase inhibitor. The *EFEMP1* gene showed lower expression in the untreated L cybrids compared to the untreated H cybrids (*p* = 0.04), but after the treatment with 5-aza-dC, the expression levels were equivalent (*p* = 0.66) because of an upregulation of *EFEMP1* in the treated L cybrids (1.78-fold, *p* < 0.033). In addition, demethylation with 5-aza-dC resulted in upregulation of *USP25*, *CXCL1*, and *CXCL8* and downregulation of *VEGFA* and *CFH* compared to the untreated cybrids. These findings suggest that the mitochondria can modulate the expression of these genes by impacting their methylation, making this process a possible target for therapies.

The *VEGFA* levels were decreased after 5-aza-dC treatment in both European-H and African-L cybrids, indicating that its transcription levels are regulated by methylation in the cybrid model. Sanchez-Navarro et al. reported that DNA hypermethylation of the VEGFA promoter gene leads to lower levels of HIF-1α/VEGFA signaling [58]. In the VEGFA promoter region, the HIF1 Binding Site (HBS) is demethylated and the HIF1α/HIF1β complex binds to the promoter to upregulation of VEGF expression [59]. Pisani et al. showed this HBS demethylation step is inhibited in aquaporin4 knock-out mice. Nashine et al. showed that demethylation treatment significantly reduced levels of VEGFA in cybrids with mitochondria from AMD patients [38]. This demonstrates the importance of the methylation status of VEGFA for angiogenesis.

Under normal conditions, the European-H and African-L cybrids expressed low levels of *CXCL1* and *CXCL8*. However, after 5-aza-dC demethylation, the *CXCL1* and *CXCL8* expression levels were increased significantly in the H cybrids, with a strong but non-significant trend in the L cybrids. CXCL1 is involved in many cellular processes such as inflammation, angiogenesis, and neutrophil recruitment, and its role in cancer has been investigated extensively [60,61]. Our findings suggest that the presence of methylation inhibits its expression and demethylation induces the CXCL levels. Zhou et al. reported that histone methylation reduces the CXCL1 expression [62]. In addition, a higher methylation status of CXCL1, among other inflammation-related genes, was found in obese subjects compared to non-obese individuals [63]. Respiratory epithelial cells producing low levels of DNMT3B expressed higher levels of CXCL1 and CXCL8, which was associated with increased neutrophil recruitment during bacterial infections [64].

The African-L cybrids showed an increased expression of *USP25* after 5-aza-dC demethylation (41.1%, *p* = 0.04) while the treated H cybrids remained unchanged. USP25 is a deubiquitinating enzyme that responds to endotoxin lipopolysaccharide (LPS) stimulation and binds to histone acetyltransferase HBO1 to modulate inflammatory gene transcription in macrophages and THP-1 monocytes [65]. This demonstrated that the mitochondrial genome from different racial/ethnic populations can play a role in the methylation status and may modulate the genes within inflammation and immune response pathways (Figure 5).

*Potential Methylation Sites in the MT-Dloop*: Recent studies have shown that differential levels of methylation at specific CpG and Non-CpG sites can account for varying occurrences in diseases such as laryngeal squamous cell carcinoma, prostate cancer, colorectal cancer, and systemic lupus erythematosus [66,67,68]. Previous studies have shown that both CpG and Non-CpG methylation sites were present in mtDNA [29]. We speculated that, since the L cybrids had different levels of total global methylation and expression of methylation-related genes, they may have different numbers of CpG and Non-CpG methylation sites compared to H and J cybrids. Analyses of the MT-Dloop regions from nucleotides 16411-460 showed that total potential methylation sites (CpG plus Non-CpG) were identical in H and J cybrids (86 sites) but varied slightly in the L cybrids (range 84–86). H and J cybrids showed 21 CpG sites for each group, while the L cybrids had a range of 21 to 22. The L cybrids had more variability in the Non-CpG sites compared to H or J cybrids. For example, the L cybrids showed two site variations in all Non-CpG sites compared to the H and J cybrids. As a result of this variability, the total number of possible methylation sites was in a range of 84 to 88 in the L cybrids. The number of potential methylation sites with the mtDNA may play a role in the different transcription levels seen in African-L cybrids compared to the European-H or European-J cybrids. In plants and fungi, the importance of Non-CpG methylation has been recognized [69,70], but it is likely also important for the regulation of methylation within the mtDNA. For example, higher levels of methylation of African-L haplogroup mtDNA may upregulate mitochondrial-encoded genes, resulting in altered signaling to the nuclear genome. However, the mechanism is not yet understood and will require additional studies.

*Acetylation Studies:* The mtDNA variants are also capable of modulating the expression levels of acetylation-related genes [40]. Furthermore, acetylation has been illustrated as a critical regulator of signaling with over 4500 acetylation sites on mitochondrial-related proteins [71]. The *HDAC* gene family is comprised of proteins that function as histone deacetylases. Histones play a crucial role in DNA interaction, blocking transcription, and maintaining nucleosome integrity. Our qRT-PCR data on acetylation-related genes were gathered on our cybrid samples. The results demonstrated that genes associated with acetylation (*HAT1*, *HDAC1*, *HDAC6*, *HDAC11*, and *SIN3A*) were similar in cybrids with African-L-origin mtDNA variants when compared with European-H-origin mtDNA variants. This is in contrast with reports of significantly lower *HAT1* and *SIN3A* levels in Ashkenazi-Jewish-K cybrids compared to European-H cybrids [30]. These variations in expression levels for the acetylation genes again demonstrate that the mitochondria representing different ancient mtDNA lineages can differentially modulate the expression patterns of critical signaling genes.

*Histone Cluster Genes:* Along with DNA methylation, epigenetics involves the acetylation and deacetylation of histones. Histones are critical in the transcription and integrity of the nucleosome. Hyland et al. (2005) showed that in *Saccharomyces cerevisiae*, a fungus known as Brewer’s yeast, certain mutations in histone subunits H3 and H4 caused hypersensitivity to hydroxyurea (HU), a DNA-damaging agent [72]. HU functions by inhibiting ribonucleotide reductase, an enzyme that catalyzes the production of deoxyribonucleotides. Furthermore, in a comparison of H3 and H4 histones between different organisms, it was shown that histone H3 exhibits a higher degree of post-translational modifications (PTMs) and acetylation pattern differences. Garcia et al. (2007) suggested that, based on this data, the histone variant H3 exhibits a higher degree of epigenetic modifications when compared to the histone variant H4 [73]. Moreover, it is the high expression of the nucleosome histone H3.3 that is associated with low-grade gliomas and may serve as a predictive marker [74]. Figure 4A depicts the sites of epigenetic modification in the N-terminus segments of the histones H3 and H4. In our samples, *HIST1H3F* expression levels were significantly higher in African-L cybrids, while expression levels for the other histone genes were similar in European-H cybrids and African-L cybrids. Upregulation of *HIST1H3F* has been reported in cutaneous squamous cell carcinoma [75]. The significance of elevated *HIST1H3F* gene expression may be found in recent studies showing *HIST1H3F* as a potential neopeptide in breast cancer [76]; a valuable prognostic marker for bladder cancer [77]; and a predictor for worse overall survival in patients with laryngeal squamous cell carcinoma [78].

In summary, since histones are critical for the regulation of chromatin and DNA compaction, and the abnormal levels of *HIST1H3F* may contribute to a decline in control of the cell cycle that is often associated with tumor growth and metastasis. we believe it is significant that cybrids containing the same nuclear DNA but either African-L or European-H mtDNA can show such varying expression levels of the *HIST1H3F* histone cluster gene. While the mechanisms by which the different racial mtDNA might signal to the nuclear DNA are still not well understood, our results may open the door for future investigations on the topic.

## 5. Conclusions

Our findings show that cybrids (Figure 5) with maternal African mitochondria (L haplogroup mtDNA) have different (i) responses to exogenous stressors (Amyβ, and UV radiation), (ii) epigenetic status, and (iii) modulation of methylation-mediated downstream complement, inflammation, and angiogenesis genes commonly associated with various human diseases than European-H haplogroup cybrids.

## Data Availability

Not applicable.

## Supplementary Materials

The following supporting information can be downloaded at: https://www.mdpi.com/article/10.3390/cells11172655/s1, Table S1: Summary of Diseases with Altered Methylation Levels in African (Afn) Versus Caucasian (Cau) Populations; Table S2: Demographics details of H and L cybrids; Table S3: Description of Genes Analyzed After UV radiation Treatment* and Inhibition with 5-aza-dC (Methylation Inhibitor); Table S4: Description of Methylation Genes; Table S5: Expression levels of genes before and after treatment with 5-aza-dC, a methylation inhibitor; Table S6: Description of Acetylation Genes; Table S7: Description of Histone Genes; Table S8: Description of Histone Genes.
