# Supplementary material for: Differential Epigenetic Status and Responses to Stressors between Retinal Cybrids Cells with African versus European Mitochondrial DNA: Insights into Disease Susceptibilities
Source: Cells. 2022 Aug 26;11(17):2655. doi: 10.3390/cells11172655 (PMC9454894; doi:10.3390/cells11172655)
Supplement: Supplementary file 1 [file cells-11-02655-s001.zip › cells-1854773-supplementary.pdf]

SUPPLEMENTARY

Supplementary Table S1: Summary of Diseases with Altered Methylation Levels in African (Afn) Versus Caucasian (Cau) Populations

DISEASE	Description	Predictor Genes	Reference
**Laryngeal Squamous Cell Carcinoma**	Aberrant methylation and race are predictors of late-stage disease outcomes	Estrogen receptor 1(ESR1)<br>Hypermethylation in cancer 1 (HIC1)	[83]
**Prostate Cancer**	Aberrant methylation contributes to the reduced expression of *OCT3* in high-grade prostate tumors<br>Genes with regulatory roles are significantly more likely to be methylated in the prostate tissue of Afn men than Cau men<br>25 novel promoter-associated CpG sites show higher methylation prevalence in Afn men than Cau men	Organic cation transporter 3 (*OCT3, SLC22A3)*<br>Androgen Receptor *(AR),* Secreted protein Acidic and Cysteine Rich *(SPARC),* Tissue Inhibitor of Metalloproteinases 3 *(TIMP3), 4)* Homeobox Protein NK-2 Homolg 5 *(NKX2-5)*	[69]<br>[70]
**Colorectal Cancer**	355 CpG sites specifically methylated in CRCs from Afn patients. CpG sites were located in 14 promoter regions of 13 genes	*ACTB*, *ATXN7L1* (ampilicon2 [79]), *BMP3, CDH5, EID3, GAS7* (amp1), *GNAS, GPR75* (amp 2), *HNRNPF* (amp 1), *NDRG4* (amp 1), Sept9, *SPAG5-AS1,* and *TNFAIP2* (amp1 and amp2).	[84]
**Systemic Lupus Erythematosus**	Significant hypo-methylation (gene enrichment) in pro-apoptotic and pro-inflammatory genes in Afn might play a role in increased frequency and severity of SLE	*TNFRSF10A, COL18A1, CDKN1A, AHRR, UNC13D, RIPK1, GRIN1, AKAP13, VAV2,* and *PRODH*<br>*IL32 (Interleukin 32)*	[71]

Supplementary Table S2: Demographics details of H and L cybrids

Cybrid	Age	Sex	Race	Haplogroup
H1 10-07	49	M	White	H66a
H2 11-10	30	M	White	H4a1a4b2
H3 11-35	30	F	White	H1b5
H4 11-23	22	F	White	H11a2a2
H5 13-49	26	M	White	H1j
H6 13-52	58	F	White	H1
H7 13-65	52	F	White	H4a1a4b
L1 11-30	54	F	Black/Multiracial	L1b2a
L2 11-31	42	M	Black	L2b2
L3 11-38	38	F	Black	L0a1a1
L4 13-124	31	M	Middle East	L1b2a
L5 13-125	52	F	Black	L1c2a1
L6 13-126	39	F	Black	L1b1a7

Supplementary Table S3. Description of Genes Analyzed After UV radiation Treatment* and Inhibition with 5-aza-dC (Methylation Inhibitor)

Symbol	Gene Name	GenBank Accession No.	Function
**_*BBC3_**	BCL2 binding component 3	NM_014417, NM_00112741, NM_001127240	Pro-apoptotic, BCL2 family, BH3. Induces permeabilization of mitochondrial outer membrane, caspase activation, mitochondrial dysfunction, and apoptosis.
**_BCL2L11_**	BCL2 like 11	NM_001204106	Anti- or pro-apoptotic regulators that are involved in a wide variety of cellular activities. Contains a Bcl-2 homology domain 3 (BH3). Interacts with other members of the BCL-2 protein family and to acts as an apoptotic activator.
**_*BLC2L13_**	BCL2-like 13	NM_015367	Over-expression leads to apoptosis. Mitochondria-specific protein.
**_*CFH_**	Complement factor H	NM_000186	Regulation of complement activation.
**_*CD55/DAF_**	Decay-accelerating factor for complement	NM_000574, NM_001114543, NM_001114544, NM_00111475	Regulates complement cascade. Accelerates decay of complement proteins.
**_*CD59_**	CD59 Molecule, Complement regulatory protein	NM_000611, NM_203329, NM_203331, NM_001127223, NM_001127225, NM_001127226, NM_001127227	Regulates complement-mediated lysis of cells. Plays a role in lymphocyte signal transduction. Blocks complement membrane attach complex.
**_CXCL1_**	C-X-C motif chemokine ligand 1	NR_046035	Antimicrobial gene; encodes a member of the CXC subfamily of chemokines. It is a secreted growth factor that signals through the G-protein coupled receptor CXC receptor 2. Plays a role in inflammation and as a chemoattractant for neutrophils.
**_CXCL5_**	C-X-C motif chemokine ligand 5	NM_002994	This gene encodes a protein that is a member of the CXC subfamily of chemokines. Chemokines, which recruit and activate leukocytes, are classified by function (inflammatory or homeostatic) or by structure. This protein is proposed to bind the G-protein coupled receptor chemokine (C-X-C motif) receptor 2 to recruit neutrophils, to promote angiogenesis and to remodel connective tissues. This protein is thought to play a role in cancer cell proliferation, migration, and invasion.

CXCL8	C-X-C motif chemokine ligand 8	NM_000584	Part of the CXC chemokine family. A major mediator of the inflammatory response. Secreted by several cell types and functions as a chemoattractant and angiogenic factor.
****EFEMP1***	EGF containing fibulin-like extracellular matrix protein	NM_001039348	Member of the fibulin family of extracellular matrix glycoproteins. Contains tandemly repeated EGF-like repeats.
****IL33***	Interleukin 33	NM_001199740, NM_033439, XM_005251626, XM_005251627	Binds to the IL1RL1/ST2 receptor. Involved in maturation of Th2 cells and activation of other leukocytes that induce inflammation.
NFKB1	Nuclear factor kappa B subunit 1	NM_003998	A transcription regulator that is activated by various intra- and extra-cellular stimuli such as cytokines, oxidant-free radicals, ultraviolet irradiation, and bacterial or viral products. Activated NFKB translocates into the nucleus and stimulates the expression of genes involved in a wide variety of biological functions.
NFKBIA	NFKB inhibitor alpha	NM_020529	Member of the NF-kappa-B inhibitor family. Interacts with REL dimers to inhibit NF-kappa-B/REL complexes which are involved in inflammatory responses. The encoded protein moves between the cytoplasm and the nucleus via a nuclear localization signal and CRM1-mediated nuclear export.
****RARA***	Retinoic acid receptor, alpha	NM_000964, NM_001145301, NM_001033603	Nuclear retinoic acid receptor involved in differentiation and apoptosis. Transcription regulator.
****TGFA***	Transforming growth factor, alpha	NM_003236, NM_001127180	Ligand for EGFR that activates a signaling pathway for differentiation, cell proliferation, and activation. Upregulated in cancers.
TRADD	TNFRSF1A associated via death domain	NM_003789	A death domain containing an adaptor molecule that interacts with TNFRSF1A/TNFR1 and mediates programmed cell death signaling and NF-kappaB activation. This protein binds adaptor protein TRAF2, reduces the recruitment of inhibitor-of-apoptosis proteins (IAPs) by TRAF2, and thus suppresses TRAF2-mediated apoptosis. This protein can also interact with receptor TNFRSF6/FAS and adaptor protein FADD/MORT1, and is involved in the Fas-induced cell death pathway.
TRAF1	TNF-receptor-associated factor 1	NM_001190945	A member of the TNF-receptor (TNFR)-associated factor (TRAF) protein family that associates with and mediates the signal transduction from various receptors of the TNFR superfamily. Forms a heterodimeric complex with TRAF2; required for TNF-alpha-mediated activation of MAPK8/JNK and

			NF-kappaB. The protein complex formed by this protein and TRAF2 also interacts with inhibitor-of-apoptosis proteins (IAPs), and thus mediates the anti-apoptotic signals from TNF receptors.
TRAF2	TNF-receptor-associated factor 2	NM_021138	Member of the TNF-receptor-associated factor (TRAF) protein family. Directly interacts with TNF receptors and forms a heterodimeric complex with TRAF1. Required for TNF-alpha-mediated activation of MAPK8/JNK and NF-kappaB. The protein complex formed by this protein and TRAF1 interacts with the inhibitor-of-apoptosis proteins (IAPs) and functions as a mediator of the anti-apoptotic signals from TNF receptors. The interaction of this protein with TRADD, a TNF-receptor-associated apoptotic signal transducer, ensures the recruitment of IAPs for the direct inhibition of caspase activation. BIRC2/c-IAP1, an apoptosis inhibitor possessing ubiquitin ligase activity, can unbiquitinate and induce the degradation of this protein, and thus potentiate TNF-induced apoptosis.
USP2	Ubiquitin-specific peptidase 2	NM_001243759	Member of the family of de-ubiquitinating enzymes. Ubiquitin-specific protease required for TNF-alpha (tumor necrosis factor alpha)-induced NF-kB (nuclear factor kB) signaling.<br>Deubiquitinates polyubiquitinated target proteins such as fatty acid synthase, murine double minute 2 (MDM2), MDM4/MDMX, and cyclin D1.
UBE3D	Ubiquitin protein ligase E3D	NM_198920, XM_006715596, XM_006715597	Ubiquitin–proteasome system important for degradation of proteins.
USP25	Ubiquitin-specific peptidase 25	NM_013396	Mediates the release of ubiquitin from degraded proteins by disassembly of the polyubiquitin chains.
USP34	Ubiquitin-specific peptidase 34	NM_014709	Mediates the release of ubiquitin from degraded proteins by disassembly of the polyubiquitin chains.
USP53	Ubiquitin-specific peptidase 53	NM_019050	Mediates the release of ubiquitin from degraded proteins by disassembly of the polyubiquitin chains.
VEGFA	Vascular endothelial growth factor A	NM_001025366, NM_001025367, NM_001025368, NM_001033756, NM_001171623, NM_001171624, NM_001171625, NM_001171626, NM_001171629,	Member of PDGF/VEGF family. Heparin-binding protein.<br>Induces proliferation and migration of vascular endothelial cells.

	NM_003376,<br>NM_001287044	

Supplementary Table S4. Expression levels of genes before and after treatment with UV radiation Treatment

Genes	Untreated (UT)			UV treatment								
	H (UT)* vs L (UT)<br>p-value<br>Mean fold change Diff.			H (UT)* vs H (UV)<br>p-value<br>Mean fold change Diff.			L (UT)* vs L (UV)<br>p-value<br>Mean fold change Diff.			H (UV)* vs L (UV)<br>p-value<br>Mean fold change Diff.		
	Time (hrs)			Time (hrs)			Time (hrs)			Time (hrs)		
	0	72	120	0	72	120	0	72	120	0	72	120
CFH	0.10<br>0.2±0.11	**<0.0001**<br>**5.22±0.19**	**<0.0001**<br>**3.43±0.14**	-	**<0.0001**<br>**1.76±0.18**	**0.004**<br>**0.63±0.17**	0.99<br>0±0.11	**0.02**<br>**0.41±0.15**	**<0.0001**<br>**1.27±0.17**	0.100<br>0.19±0.11	**<0.0001**<br>**3.05±0.1**	**<0.0001**<br>**1.53±0.2**
CD55	**<0.0001**<br>**-0.88±0.11**	**<0.001**<br>**0.30±0.06**	0.07<br>-0.25±0.13	-	0.47<br>0.11±0.14	0.37<br>0.09±0.1	0.99<br>0±0.08	**0.02**<br>**-0.56±0.21**	**<0.0001**<br>**-**<br>**1.41±0.17**	**<0.0001**<br>-0.89±0.06	0.55<br>0.15±0.25	**<0.0001**<br>**-1.58±0.15**
CD59	0.40<br>0.13±0.14	**0.001**<br>**-0.34±0.07**	**<0.0001**<br>**0.48±0.05**	-	0.22<br>0.10±0.08	0.29<br>-0.09±0.08	0.99<br>0±0.15	**0.001**<br>**0.31±0.07**	0.73<br>0.02±0.05	**0.03**<br>**0.13±0.05**	0.36<br>0.08±0.08	**<0.0001**<br>**0.58±0.07**
IL-33	**<0.0001**<br>**0.77±0.02**	**<0.0001**<br>**-1.18±0.14**	**0.002**<br>**-0.45±0.11**	0.34<br>0.13±0.13	**0.003**<br>**0.73±0.19**	**0.0003**<br>**0.55±0.19**	0.92<br>0.0±0.03	**0.001**<br>**0.51±0.09**	**<0.0001**<br>**0.67±0.10**	**0.02**<br>**0.63±0.15**	**0.0002**<br>**-0.96±0.16**	**0.0002**<br>**-0.57±0.09**
TGF-A	**0.002**<br>**0.36±0.09**	**0.001**<br>**-0.87±0.17**	**<0.0001**<br>**-1.22±0.18**	-	**0.008**<br>**0.38±0.11**	**0.001**<br>**0.72±0.15**	0.99<br>0.0±0.103	**0.009**<br>**0.62±0.19**	**0.0005**<br>**0.84±0.17**	**<0.0001**<br>**0.36±0.05**	**0.0002**<br>**-**<br>**0.96±0.16**	**<0.0001**<br>**-1.34±0.13**
EFEMP1	**<0.0001**<br>**0.31±0.05**	0.09<br>0.20±0.11	0.368<br>**-0.07±0.07**	-	**0.006**<br>**0.37±0.11**	**<0.0001**<br>**1.02±0.08**	0.99<br>0.0±0.06	**0.001**<br>**0.34±0.07**	**<0.0001**<br>**0.47±0.04**	**<0.0001**<br>**0.31±0.05**	**0.008**<br>**0.23±0.07**	**<0.0001**<br>**0.62±0.05**
RARA	**0.001**<br>**0.14±0.03**	0.87<br>-0.02±0.12	0.29<br>0.09±0.08	-	0.054<br>0.22±0.10	**0.0002**<br>**0.52±0.09**	0.82<br>0.0±0.06	0.056<br>0.26±0.12	**<0.0001**<br>**0.65±0.08**	0.062<br>0.12±0.06	0.52<br>-<br>0.06±0.10	0.632<br>-0.05±0.10
BBC3	**<0.0001**<br>**0.29±0.02**	**0.04**<br>**-0.50±0.22**	**<0.0001**<br>**-0.45±0.06**	-	**0.0001**<br>**1.01±0.17**	**<0.0001**<br>**1.57±0.10**	0.99<br>0.0±0.03	0.194<br>0.32±0.23	**0.004**<br>**0.40±0.11**	**<0.0001**<br>**0.29±0.02**	0.335<br>0.18±0.18	**<0.0001**<br>**1.57±0.10**
BCL2L13	0.19<br>0.08±0.05	0.081<br>-0.50±0.22	**0.001**<br>**0.32±0.07**	-	**<0.0001**<br>**0.40±0.04**	**<0.0001**<br>**0.56±0.07**	0.99<br>0.0±0.06	0.101<br>0±0.23	**<0.0001**<br>**0.52±0.08**	0.193<br>0.08±0.06	**0.002**<br>**0.19±0.04**	**0.002**<br>**0.36±0.09**

Supplementary Table S5: Description of Methylation Genes

Symbol	Gene Name	GenBank Accession No.	Function
MAT2B	Methionine adenosyl-transferase II, beta	NM_013283	Catalyzes the biosynthesis of S-adenosylmethionine and ATP. Regulatory beta subunit.
MBD4	Methyl-CpG-binding domain protein 4	NM_003925	Binds specifically to methylated DNA. Involved in protein interactions and DNA repair.
DNMT1	DNA (cystosine-5) methyltransferase 1	NM_001130823 NM_001379	Methylates CpG residues; preferentially methylates hemimethylated DNA. Associates with DNA replication sites in S phase, maintaining the methylation pattern in the newly synthesized strand. Essential for epigenetic inheritance. Adds methylation to cytosine 5 (5mC).
DNMT3A	DNA (cystosine-5) methyltransferase 3B	NM_022552 NM_153759 NM_175629	Methylates de novo during development. Important for genomic imprinting.
DNMT3B	DNA (cystosine-5) methyltransferase 3A	NM_001207055 NM_001207056 NM_006892 NM_175848 NM_175849 NM_175850	Methylates de novo during development. Important for genomic imprinting.

Supplementary Table S6: Expression levels of genes before and after treatment with 5-aza-dC, a methylation inhibitor

Symbol	H* vs. L (Untreated) p-value Fold	H* vs L (5-aza-dC) p-value Fold	H (Untreated)* vs H (5-aza-dC) p-value Fold	L (Untreated)* vs L (5-aza-dC) p-value Fold
BCL2L11	0.82<br>0.99 ± 0.20	0.32<br>0.76 ± 0.17	0.12<br>1.81 ± 0.27	0.36<br>1.45 ± 0.31
CFH	0.17<br>0.68 ± 0.13	0.87<br>0.97 ± 0.17	**0.018**<br>0.4 ± 0.03	0.11<br>0.59 ± 0.1
CXCL1	0.49<br>2.81 ± 1.55	0.87<br>1.13 ± 0.71	**0.0002**<br>363.9 ± 25.84	0.19<br>408.1 ± 257.4
CXCL5	0.35<br>9.33 ± 7.19	0.87<br>1.07 ± 0.8	0.11<br>4554 ± 2212	0.25<br>1191 ± 889.3
CXCL8	0.74<br>3.3 ± 2.8	0.98<br>41.4 ± 20.8	**0.03**<br>42.03 ± 11.9	0.14<br>31.24 ± 15.7
EFEMP1	**0.04**<br>0.75 ± 0.07	0.66<br>0.93 ± 0.12	0.13<br>1.46 ± 0.24	**0.033**<br>1.78 ± 0.22
NFKB1	0.78<br>1.01 ± 0.12	0.7<br>0.94 ± 0.11	0.5<br>1.53 ± 0.45	0.16<br>1.35 ± 0.156
NFKBIA	0.41<br>2.17 ± 0.92	0.4<br>3.37 ± 1.44	0.53<br>1.84 ± 0.78	0.52<br>3.37 ± 1.44
TRAF1	0.45<br>4.27 ± 3.14	0.36<br>2.8 ± 1.43	0.33<br>3.24 ± 1.27	0.56<br>4.18 ± 2.14
TRAF2	0.27<br>1.96 ± 0.7	0.25<br>1.89 ± 0.62	0.9<br>0.998 ± 0.21	0.95<br>1.89 ± 0.62
USP2	0.78<br>0.89 ± 0.4	0.59<br>1.29 ± 0.49	0.34<br>0.89 ± 0.06	0.67<br>1.56 ± 0.59
UBE3D	0.22<br>1.47 ± 0.31	0.11<br>1.39 ± 0.25	0.26<br>0.81 ± 0.12	0.85<br>1.39 ± 0.25
USP25	0.3<br>0.79 ± 0.04	0.85<br>1.11 ± 0.1	0.94<br>1.06 ± 0.23	**0.04**<br>1.41 ± 0.13
USP34	0.27<br>1.47 ± 0.34	0.1<br>2.2 ± 0.56	0.4<br>0.89 ± 0.07	0.48<br>1.4 ± 0.36

USP53	0.68<br>1.09 ± 0.19	0.051<br>1.65 ± 0.19	0.9<br>1.03 ± 0.14	0.095<br>1.56 ± 0.18
VEGFA	0.12<br>0.79 ± 0.07	0.45<br>1.1 ± 0.11	**0.003**<br>0.45 ± 0.04	**0.029**<br>0.64 ± 0.06
**Symbol**	**H* vs. L (Untreated)<br>p-value<br>Fold**	**H* vs L (5-aza-dC)<br>p-value<br>Fold**	**H (Untreated)* vs H (5-aza-dC)<br>p-value<br>Fold**	**L (Untreated)* vs L (5-aza-dC)<br>p-value<br>Fold**

Fold values greater than 1 indicate up-regulation of the gene compared to H cybrids.
Fold values less than 1 indicate down-regulation of the gene compared to H cybrids.
H cybrids are assigned a value of 1.
Fold = $2^{\Delta\Delta CT}$
*n = 3 different H cybrids and n = 3 different L cybrids, with three values for each sample.

Supplementary Table S7: Description of Acetylation Genes

Symbol	Gene Name	GenBank Accession No.	Function
HAT1	Histone acetyltransferase	NM_001033085 NM_003642	Adds acetyl group.
HDAC1	Histone deacetylase 1	NM_004964	Class I histone deacetylase. Found primarily in nucleus. Involved in control of cell proliferation, differentiation, growth, and apoptosis.
HDAC6	Histone deacetylase 6	NM_006044	Class IIB histone deacetylase. Cytoplasmic, microtubule-associated. Deacetylates tubulin, Hsp90, cortactin.
HDAC11	Histone deacetylase 11	NM_024827	Class IV histone deacetylase. Localized to the nucleus. Involved in regulating interleukin 10 expression.
SIN3A	Sin3 transcription regulator homolog A (yeast)	NM_001145357 NM_001145358 NM_015477	Transcriptional regulator protein. Forms complex with histone deacetylases HDAC1 and HDAC2. Promotes deacetylation.

Supplementary Table S8: Description of Histone Genes

Symbol	Gene Name	GenBank Accession No.	Function
HIST1H3A	Histone cluster 1, H3a	NM_003529	Basic nuclear proteins responsible for the nucleosome structure of the chromosomal fiber in eukaryotes.<br>Gene lacks introns. Encodes a member of the histone H3 family. Transcripts lack polyA tails.<br>Contain a palindromic termination element.
HIST1H3C	Histone cluster 1 H3 family member c	NM_003531	Gene lacks introns. Encodes a replication-dependent histone that is a member of the histone H3 family.<br>Transcripts lack polyA tails.<br>Contains a palindromic termination element.
HIST1H3F	Histone cluster 1, H3f	NM_021018	Gene lacks introns. Encodes a replication-dependent histone that is a member of the histone H3 family.<br>Transcripts from this gene lack polyA tails.<br>Contains a palindromic termination element.
HIST1H3H	Histone cluster 1, H3h	NM_003536	Gene lacks introns. Encodes a replication-dependent histone that is a member of the histone H3 family.<br>Transcripts lack polyA tails.<br>Contains a palindromic termination element.
HIST1H3J	Histone cluster 1 H3 family member j	NM_003535	Gene lacks introns. Encodes a replication-dependent histone that is a member of the histone H3 family.<br>Transcripts lack polyA tails.<br>Contains a palindromic termination element.
HIST1H4H	Histone cluster 1, H4h	NM_003543	Gene lacks introns. Encodes a member of the histone H4 family. Transcripts lack polyA tails.<br>Contains a palindromic termination element.
